# Resistome in Lake Bolonha, Brazilian Amazon: Identification of Genes Related to Resistance to Broad-Spectrum Antibiotics

**DOI:** 10.3389/fmicb.2020.00067

**Published:** 2020-02-04

**Authors:** Jorianne Alves, Larissa Dias, Jackeline Mateus, Joana Marques, Diego Graças, Rommel Ramos, Lucy Seldin, Isabel Henriques, Artur Silva, Adriana Folador

**Affiliations:** ^1^Laboratório de Genômica e Bioinformática, Centro De Genômica e Biologia de Sistemas, Universidade Federal Do Pará, Belém, Brazil; ^2^Instituto de Microbiologia Paulo de Góes, Universidade Federal do Rio de Janeiro, Rio de Janeiro, Brazil; ^3^Center for Environmental and Marine Studies (CESAM), University of Aveiro, Aveiro, Portugal; ^4^Department of Life Sciences, Faculty of Science and Technology, University of Coimbra, Coimbra, Portugal

**Keywords:** metagenome, Lake Bolonha, resistome, bacteria resistant to antibiotics, β-lactams

## Abstract

Resistance to antibiotics is one of the most relevant public health concerns in the world. Aquatic environments play an important role because they are reservoirs for antibiotic resistance genes and antibiotic-resistant strains, contributing to the spread of resistance. The present study investigated the resistome in Lake Bolonha (three sampling sites) in the Amazon region using a metagenomics approach and culture-dependent methods. Whole-metagenome-based results showed that the most abundant phyla were Protobacteria, Actinobacteria, Firmicutes, Bacteroidetes and Cyanobacteria. The composition of the resistome demonstrated that the genes that confer resistance to β-lactams were prevalent at all sampling sites, followed by genes conferring resistance to aminoglycosides and tetracycline. Acquired genes encoding extended-spectrum β-lactamases (e.g., *bla*_CTX–M_) and resistance to carbapenems (e.g., *bla*_IMP_ and *bla*_VIM_) were detected through metagenome analysis. Bacteria were isolated from culture medium supplemented with cefotaxime or imipenem, and isolates were identified and analyzed for their antibiotic susceptibility profiles and resistance genes. In total, 98 bacterial isolates belonging to the genera *Pseudomonas* (37), *Acinetobacter* (32), *Klebsiella* (13), *Enterobacter* (9), *Pantoe* (3), *Stenotrophomonas* (3), and *Methylobacterium* (1) were obtained. Among isolates, the most abundant genes were *bla*_CTX–M_ (28.3%), *bla*_SHV_ (22.6%) and *bla*_TEM_ (18.8%) in isolates from cefotaxime-supplemented medium and *bla*_VIM_ (28.8%) and *bla*_IMP_ (22.2%) in isolates recovered from imipenem-supplemented medium. The genes *intl*1 and *intl*2 were detected in 19.3% and 7.1% of isolates. Antibiograms showed that 94.9% (from cefotaxime-supplemented medium) and 85.7% (from imipenem-supplemented medium) of the isolates were multidrug resistant. Besides cefotaxime and imipenem, isolates were mostly resistant to aztreonam (91.8%), amoxicillin (98.8%), ampicillin (82.6%), and nalidixic acid (77.5%). Hence, the present study demonstrates that Lake Bolonha is a reservoir of bacteria resistant to antibiotics and resistance genes, some of which are of critical importance to human health.

## Introduction

Antibiotic resistance is considered by the World Health Organization to be one of the world’s three greatest threats to human health (World Health Organization [WHO], 2014) because of the extensive spread of antibiotic-resistant bacteria (ARB) and antibiotic resistance genes (ARGs).

Antibiotic-resistant bacteria and ARGs have been reported in the environment, such as in soil (Cadena et al., 2018; Djenadi et al., 2018) and aquatic systems (Tacão et al., 2012; Li et al., 2018; Obayiuwana et al., 2018). Antibiotics and resistant bacteria are released into the environment through wastewater effluents and agricultural and livestock flows, altering natural ecosystems and microbial population dynamics, as well as introducing selective pressure that contributes to the diversity of the ARG pool. Therefore, aquatic environments are considered the main reservoirs of ARGs and ARB (Coque et al., 2008; Wright, 2010; Tacão et al., 2012).

Inadequate water treatment and lack of basic sanitation are factors that can increase resistance to antibiotics, promoting ARB spread and the exchange of genetic material between bacteria (Osiñska et al., 2016a). Many opportunistic pathogenic microorganisms resistant to antibiotics can proliferate in the environment, such as *Pseudomonas*, *Stenotrophomonas*, *Acinetobacter*, and *Burkholderia*, carrying ARGs, some of which are encoded in mobile genetic elements (Wright, 2010). Thus, studies in aquatic environments seek to identify the prevalence of ARB and to correlate their presence with the horizontal transfer of ARGs (Osiñska et al., 2016b; Karkman et al., 2018; Obayiuwana et al., 2018).

Metagenomic approaches have been applied to understand and monitor the mechanisms of resistance and their evolution in microbial communities and are considered important tools for the study of microbial ecology (Karkman et al., 2018). In addition, culture-dependent methods allow the isolation of target bacteria to study their phenotypic and genotypic characteristics related to antibiotic resistance (McLain et al., 2016). The application of these associated methodologies allows the characterization of the microbiome and resistome, such as in the Brazilian Amazon region, which, despite having the largest hydrographic basin in the world, has rarely been addressed concerning resistance to antibiotics (de Lima et al., 2016; Freitas et al., 2019).

Hence, this study aims to characterize the bacterial community and antibiotic resistance in Lake Bolonha, one of the main sources of water in the metropolitan region of Belém-Pará, Brazil. As described for other aquatic systems, anthropogenic activities may result in an altered bacterial community, with a higher prevalence of antibiotic resistant bacteria and resistance genes. To confirm this hypothesis a metagenomic approach and culture-dependent methods were applied to identify the presence of ARGs and ARB, as well as to monitor the environment for its potential role in the spread and evolution of antibiotic resistance. In terms of culture-dependent analyses, a special focus was put on resistance to β-lactams, which are among the most commonly used antibiotics. Clinically relevant β-lactams include 3rd-generation cephalosporins (e.g., cefotaxime) and carbapenems (e.g., imipenem). These are widely used to treat infections caused by important pathogens, such as *Escherichia coli*, *Salmonella enterica*, and *Klebsiella pneumoniae*, which cause a variety of diseases in humans and animals (Nordmann and Cornaglia, 2012; D’Andrea et al., 2013; Djenadi et al., 2018). Carbapenems are used as last resort drugs to treat infections caused by multidrug resistantbacteria (Tacão et al., 2015).

## Materials and Methods

### Water Sampling

**FIGURE 1:**
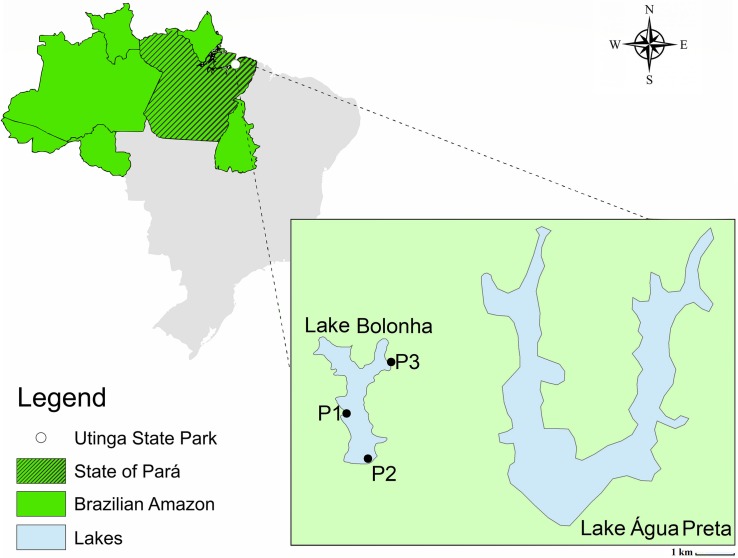
Map of Lake Bolonha with the sampling sites marked.

The water samples were collected in January 2017 from Lake Bolonha, Belém, Pará. Three sites were selected along the lake: site 1 - water catchment area from Lake Bolonha to the Water Treatment Station (S 01°25.530^″^ W 048°26.043^″^); site 2 - local evacuation of water from the lake to other treatment substations (S 01°25.530^″^ W 048°26.018^″^); site 3 - channel connecting the lakes Água Preta and Bolonha (S 01°24.992^″^ W 048°25.785^″^) (Figure 1). At each sampling site, the 5 L surface water (1 m) was collected in sterile polypropylene flasks (1 L) and transported in an ice box to the laboratory, where 1 L was used for bacterial cultivation, 1 L was used in triplicate for metagenomic analysis and 1 L was used to analyze water quality.

The water quality was determined by assessing physical, chemical and microbiological parameters (Supplementary Table S1). The analysis was performed in the Faculty of Sanitary and Environmental Engineering laboratory, Federal University of Pará, Brazil, according to the procedures and recommendations described in the Standard Methods for the Examination of Water and Wastewater (APHA/AWWA/WEF, 2012). The resolution no. 357/2005 of the Environment National Council of Brazil (Brasil, 2005) was used as reference to evaluate the results.

### Metagenomics-Based Approach

#### Total Community DNA (TC-DNA) Extraction and PCR-DGGE

The water samples (1 L) from each site were collected in triplicate and filtered through 0.22 μm nitrocellulose filters (Millipore, Billerica, MA, United States). Total community DNA (TC-DNA) was extracted from the filter membrane using the UltraClean^TM^ Soil DNA kit (MoBio, EUA) according to the manufacturer’s instructions. The Qubit fluorometer (Life Technologies, Carlsbad, CA, United States) was used to measure the quantity of the TC-DNA obtained. The TC-DNA was used for 16S rRNA gene amplification with the primers U968F (5′-AAC GCG AAG AAC CTT AC-3′) containing a GC clamp (5′-CGC CCG CCG CGC GCG GCG GGC GGG GCG GGG GCA CGG GGG G-3′) and L1401R (5′ GCG TGT GTA CAA GAC CC 3′) according to Nübel et al. (1996). A negative control was included in PCR analysis, replacing the template DNA by sterilized water. PCR products and standard bacterial markers were used in denaturing gradient gel electrophoresis (DGGE) (46.5–60% of urea and formamide) according to Heuer et al. (1997). The electrophoresis parameters and silver staining of the gels were performed according to Gomes et al. (2005) and Heuer et al. (2001), respectively. The cluster analysis was performed by the unweighted pair group method with average linkages (UPGMA) using the software package GelCompar II 4.5 (Applied Maths, Ghent, Belgium). The dendrograms were constructed based on the Pearson correlation indices. The statistical analysis (*p* ≤ 0.05) of the DGGE profiles entailed three different methods: PERMTEST software, which was based on the pairwise Pearson correlation indices (Kropf et al., 2004); principal component analysis (PCA); and PERMANOVA.

#### Metagenome Community Analysis

The TC-DNA was submitted to sequencing by the Ion Proton^TM^ platform chip P1 according to the manufacturer’s protocol. After sequencing, the quality of raw data was analyzed using the FastQC tool^1^, and then the bases that presented the quality value below Phred 20 were trimmed and filtered using the FastX-Toolkit program^2^, in which reads with a minimum length of 75 bp remained. Sequences were annotated using MG-RAST (Meta Genome Rapid Annotation using Subsystem Technology). Metagenomic SEED viewer was used to predict functional abundance and to determine bacterial relative abundance at the phylum, family and genus levels. The Silva database (version 111) was used (Meyer et al., 2008) with the following parameters: e-value 10e-05, identify ≥60% and length ≥15.

#### Metagenome-Based Analyses of the Resistome

The metagenome data were submitted to the GCSplit to be assembled into contigs (Miranda et al., 2018). The coding sequences (CDSs) were predicted using Prodigal (Hyatt et al., 2010). The contigs were submitted to the CARD database (Comprehensive Antibiotic Resistance Database) to detect ARGs, based on homology against 2383 reference sequences, through the Resistance Gene Identifier (RGI) tool, using the perfect and strict algorithm (Jia et al., 2016). The programs were executed using the default parameters.

### Culture-Dependent Analyses

#### Bacteria Isolation and Identification

The water samples were filtered in triplicate (0.45 μm pore membranes – Millipore Ind. and Commerce LTDA, Brazil), and the membranes were placed on MacConkey agar (Kasvi) supplemented with cefotaxime (8 μg/ml) or imipenem (4 μg/ml). The plates were incubated for 24 h at 37°. The individual colonies were stored at −80°C in MacConkey medium with 25% (v/v) glycerol.

The genomic DNA of the isolates was extracted by the phenol-chloroform isoamyl alcohol method, according to a previously reported protocol (Wilson, 2001). A NanoDrop (ND-2000c-Thermo Scientific, United States) was used to measure the quality and quantity of genomic DNA. The 16S rRNA gene was amplified according to Massol-Deya et al. (1995). Thus, the universal primers 8F (5′-AGAGTTTGATCCTGGCTCAG-3′) and 1492R (5′-TACGGYTACCTTGTTACGACTT-3′) was used, which contained the total volume 50 μL reaction mixtures including buffer 1×, 1.5 mM of MgCl_2_, 0.2 pmol of each primer, 0.2 mM of dNTP, 1 U of Taq DNA polymerase (Invitrogen) and 50–100 ng of DNA. The cycling conditions used were: an initial denaturation at 95°C for 5 min, then 35 cycles of 95°C for 1 min, 55°C for 1 min and 72°C for 1 min, and a final extension step of 72°C for 10 min. After that, the gene was sequenced (ABI 3130 DNA Analyzer platform, Thermo Fisher Scientific), and the sequences were compared against the GenBank database using the Blast-N tool^3^ (Altschul et al., 1997).

#### Isolates Antibiotic Resistance Profiles

Genotypic and phenotypic resistance profiles of isolates were determined using a PCR approach (ARGs and integrases molecular detection) and the Kirby and Bauer method (Bauer et al., 1966), respectively. The PCR technique was applied to identify the presence of genes encoding β-lactamases (*bla*_TEM_, *bla*_VIM_, *bla*_IMP_, *bla*_CTX_, *bla*_SHV_, and *bla*_KPC_) as previously described by Alves et al. (2014) (Supplementary Table S2). To determine susceptibility profiles, 16 antibiotics were tested by the Kirby and Bauer method on Mueller-Hinton agar medium (Kasvi) in duplicate. The isolates were incubated for 16–24 h at 37°C. The antibiotics used were amoxicillin (10 μg), amoxicillin/clavulanic acid (20 μg/10 μg), ampicillin (10 μg), aztreonam (30 μg), cefepime (30 μg), ceftazidime (30 μg), cefotaxime (30 μg), cephalothin (30 μg), ciprofloxacin (5 μg), chloramphenicol (30 μg), gentamicin (10 μg), imipenem (10 μg), kanamycin (30 μg), nalidixic acid (30 μg), sulfamethoxazole/trimethoprim (25 μg) and tetracycline (30 μg) (Laborclin, Brazil). The strain *Escherichia coli* ATCC 25922 was used as a control. The mean and standard deviation of inhibition halo values of duplicate were compared to those standardized by the Clinical and Laboratory Standards Institute (CLSI) to classify the strains as susceptible, intermediate or resistant (CLSI, 2017).

## Results

### Physical, Chemical and Microbiological Analysis

From the results of the physical, chemical and microbiological analysis of Lake Bolonha water (Supplementary Table S1), values diverging from the reference values of the CONAMA resolution were observed for the concentration of total nitrogen, concentration of total phosphorus, electrical conductivity, pH and total coliforms. The total nitrogen concentration was above the reference value (1.27 mg N/L) in sites P2 (1.4 mg N/L) and P3 (1.3 mg N/L). The total phosphorus was 0.25 (P1), 0.47 (P2) and 0.35 mg P/L (P3), and the electrical conductivity was 0.134 (P1), 0.14 (P2) and 0.12 μS⋅cm (P3), while the reference values for these parameters were ≤0.030 mg P/L and ≤0.100 μS⋅cm, respectively. The total coliform counts were also above the reference value (≤3 Log10 - CFU ml^–1^) at site P1 (3.38), P2 (3.52), and P3 (3.38). The pH values were below the reference range (6.0–9.0) at three sampling sites (P1–5.9; P2–5.1; P3–4.9). Values determined for the remaining parameters were in accordance with the values defined by the CONAMA resolution.

### DGGE Analysis of Bacterial Communities

**FIGURE 2:**
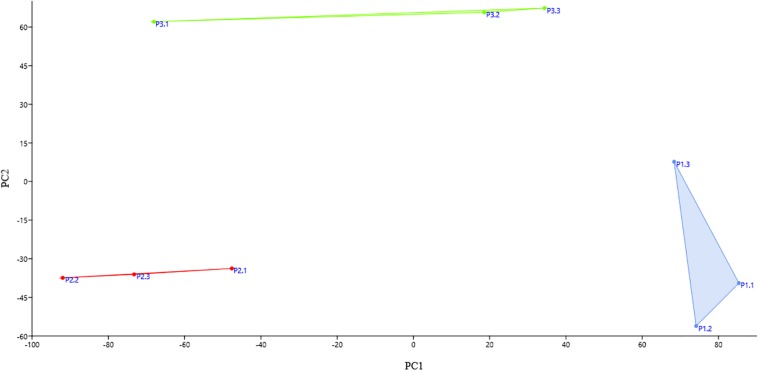
Principal component analysis (PCA) score plot of each replicate at each collection site. The replicates of the same site group together and each site’s grouping was different from the others among the three sampling sites. Blue: the replicates of site P1; red: the replicates of site P2; and green: the replicates of site P3.

The bacterial community of three replicate samples for each sampling site was analyzed using DGGE fingerprints. The results showed that the replicates of each site formed isolated clusters, corresponding to a similarity equal to or greater than 94% (Supplementary Figure S1). Principal component analysis (PCA) corroborates this result. In Figure 2, it can be observed that the replicates of the same site were grouped together in defined clusters, and each site’s grouping was different from the others among the three sampling sites. However, according to the PERMANOVA one-way test, there were no statistically significant differences (*p* ≤ 0.05) between sites.

### Bacterial Community Analysis by Metagenomics

The metagenome of the Lake Bolonha communities yielded approximately 10 million paired reads in sites P1, P2, and P3. MG-RAST identified approximately 2 million protein features in P1 and P3 and approximately 1 million in P2, and it identified 11,286, 4,459, and 9,813 ribosomal RNA gene sequences in sites P1, P2, and P3, respectively (Table 1). The data were deposited in the NCBI SRA database under the accession numbers SRR8893560 (site P1), SRR8893561 (site P2) and SRR8893559 (site P3), and in the MG-Rast under the accession numbers mgs680613 (site P1) mgs596390 (site P2), and mgs600228 (site P3).

**TABLE 1:** Sequence-read statistics from metagenome data obtained from each sampling site (P1, P2, and P3).

Parameter	P1	P2	P3
Base pair count (bp)	2,067,531,653	2,009,708,988	1,536,409,301
Total read count (bp)	10,538,522	10,190,323	10,103,487
Mean read length (bp)	153 ± 36	152 ± 37	152 ± 35
Mean GC percentage (%)	52 ± 8	56 ± 6	55 ± 7
Alignment-identified protein features	2,587,226	1,026,444	2,015,684
Alignment-identified rRNA features	11,286	4,459	9,813
Alignment-identified functional categories	2,225,603	635,898	1,783,315
α-Diversity	572	41	319
Rarefaction curve	9139	4978	7529
N50	845	4,240	874
Bases	93,290,470	37,780,414	57,491,071
Contigs	110,347	21,905	66,694

**FIGURE 3:**
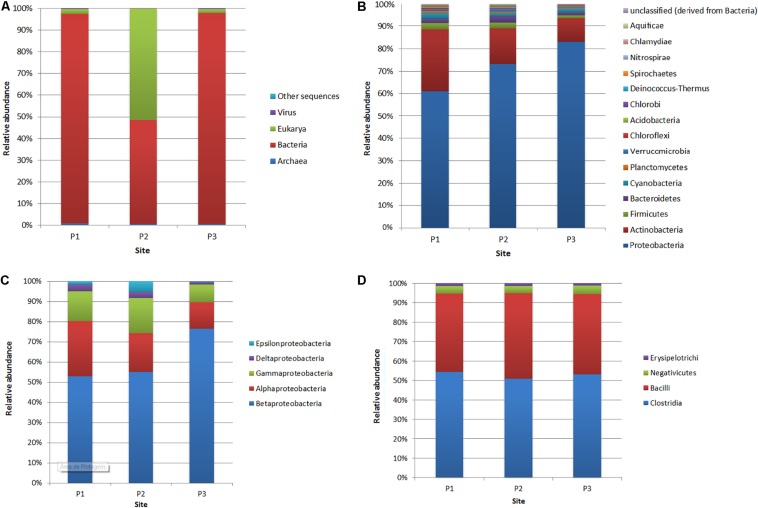
Relative abundance of reads affiliated with domains **(A)**, bacterial phyla **(B)**, Proteobacteria classes **(C)** and Firmicutes classes **(D)** at the three sites (P1, P2, and P3).

In this study, the rarefaction curves revealed that the P1 site presented greater sample richness than in the other two sites (Supplementary Figure S2). In addition, principal coordinate analysis (PCoA) has shown that locations P1 and P3 are closest to each other (Supplementary Figure S3). The domain Bacteria was predominant in P1 (96.6% of the total number of reads affiliated with the 16S rRNA gene) and P3 (97.3%), while from the P2 site, a higher number of sequences affiliated with the Eukarya domain (51.6%) was obtained, followed by Bacteria-affiliated reads (47.8%) (Figure 3A and Supplementary Figure S4). Among the Bacteria reads, the phylum most abundant in all samples was Proteobacteria (P1: 60.6% of the reads affiliated with domain Bacteria; P2: 72.8%; P3: 82.8%), followed by Actinobacteria (P1: 27.3%; P2: 15.7%; P3: 10.5%), Firmicutes (P1: 2.6%; P2: 2.5%; P3: 1.2%), Bacteroidetes (P1: 2.3%; P2: 2.8%; P3: 1.2%) and Cyanobacteria (P1: 2.3%; P2: 1.4%; P3: 1.7%) (Figure 3B).

Within the phylum Proteobacteria (Figure 3C), at site P1, the most abundant classes were Betaproteobacteria (52.8% of the 16S rRNA gene reads assigned to Proteobacteria), Alphaproteobacteria (27.3%), Gammaproteobacteria (14.5%), Deltaproteobacteria (3.2%) and Epsilonproteobacteria (1.7%). At site P2, the most abundant class was also Betaproteobacteria (54.7%), followed by Alphaproteobacteria (19.3%), Gammaproteobacteria (17.2%), Epsilonproteobacteria (5.4%) and Deltaproteobacteria (2.9%). Concerning site P3, the class Betaproteobacteria was more abundant, with 76.3% compared to other sites. However, the classes Alphaproteobacteria (13.1%), Gammaproteobacteria (8.6%) and Deltaproteobacteria (1.3%) were less abundant compared to sites P1 and P2.

Among the Betaproteobacteria, the most abundant order was Burkholderiales at all collection sites (P1: 54.9% of the reads assigned to Betaproteobacteria; P2: 74.4%; P3: 71.9%), and the Comamonadaceae family represented 54% (P1), 58.5% (P2), and 68.1% (P3) of the total abundance of this order. Within Alphaproteobacteria, the most abundant order was Rhizobiales, with 31.6% (P1), 27.6% (P2), and 33.2% (P3). At site P1, the most representative family was Methylocystaceae (41.5%), while at sites P2 and P3, the most abundant family was Bradyrhizobiaceae, with 27.6 and 33.2%, respectively.

The Actinobacteria phylum was the second most representative, with all sequences belonging to the Actinobacteria class. Thus, the most abundant order at all sampling sites was Actinomycetales, with 95.1, 95.3, and 94.9% relative abundance at sites P1, P2, and P3, respectively. Within this order, the most abundant genera were *Mycobacterium*, with 15.1, 6.5, and 7.8% at sites P1, P2, and P3, respectively, and *Streptomyces*, with 10.3, 17.6, and 15.2% at sites P1, P2, and P3.

The most predominant class in the Firmicutes phylum, considering all sites, was Clostridia, which made up more than 50% of the reads affiliated with this phylum, followed by the Bacilli class, with more than 40% abundance at the three sites (Figure 3D). The class Negativicutes presented relative abundances of 4.1% at site P1, 3.8% at site P2, and 4.4% at site P3, and the Erysipelotrichi class presented 1.2, 1.3, and 1.2% abundances, respectively, at sites P1, P2, and P3.

At site P2, a greater abundance of the Eukaryota domain with dominance of the Ascomycota phylum (99%) was demonstrated. The most representative families were Clavipitaceae and Cordycipitaceae, both belonging to the order Hypocreales of the class Sordariomycetes. Regarding Clavipitaceae, the most representative genera were *Paecilomyces* (37.4%) and *Metacordyceps* (32.7%), where *Paecilomyces marquandii* and *Metacordyceps liangshanensis* were the most abundant species. The diversity of site P2 differed from that at sites P1 and P3, where the family Nectriaceae had 99% abundance.

The most abundant 10 genera of bacteria at the three sites are described in Table 2. *Synechococcus* was the most abundant at site P1 (3.9%) and *Acidovorax* was the most abundant genus at sites P2 (5.8%), and P3 (10.9%). However, it was observed that at site P1, 41.2% of the sequences were not classified at the genus level.

**TABLE 2:** The relative abundance of the ten most abundant genera of bacteria in each site.

P1	%	P2	%	P3	%
*Synechococcus*	3.9	*Acidovorax*	5.8	*Acidovorax*	10.9
Unclass. *Epsilonproteobact.* genus	3.7	*Polaromonas*	3.9	*Dechloromonas*	9.8
Unclass. *Betaproteobact.* genus	3.3	*Burkholderia*	2.6	*Polaromonas*	6.8
*Mycobacterium*	2.5	*Streptomyces*	2.6	*Albidiferax*	3.9
Unclass. *Alphaproteobact.* genus	1.9	*Albidiferax*	2.2	*Burkholderia*	3.1
*Streptacidiphilus*	1.8	*Polynucleobacter*	2.0	*Delftia*	2.1
Unclass. *Proteobact.* genus	1.8	*Pseudomonas*	1.6	*Variovorax*	2.0
*Streptomyces*	1.7	*Methylobacillus*	1.5	*Comamonas*	1.8
*Terrabacter*	1.4	*Dechloromonas*	1.4	*Polynucleobacter*	1.8
*Methylomonas*	1.3	*Cupriavidus*	1.4	*Verminephrobacter*	1.7

**FIGURE 4:**
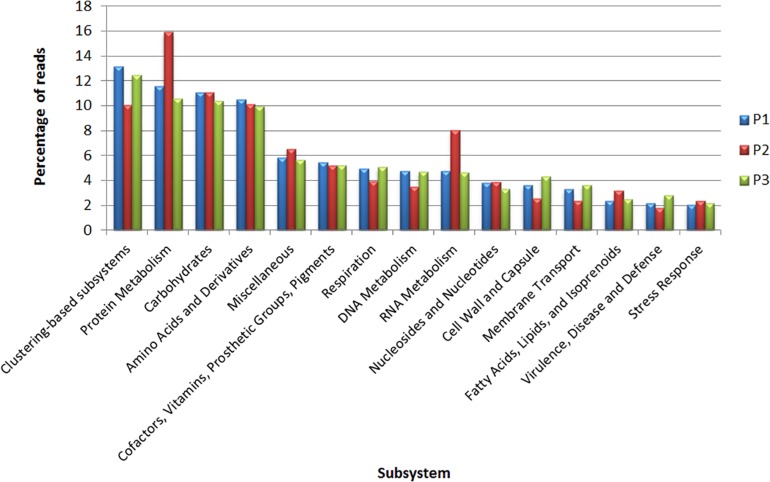
Classification of metagenomic data obtained from sites P1, P2, and P3 in subsystems according to MG-RAST.

The subsystem analysis was also performed for each site through the MG-RAST (Figure 4). The four most represented subsystems were clustering-based subsystems (P1: 13.1%; P2: 10%; P3: 12.4%), protein metabolism (P1: 11.5%; P2: 15.9%; P3: 10.5%) carbohydrates (P1: 11.1%; P2: 11%; P3: 10.3%) and amino acids and derivatives (P1: 10.5%; P2: 10.1%; P3: 9.9%). Regarding the genes related to virulence and defense, for each site, the relative abundances were P1: 2.1%; P2: 1.7%; and P3: 2.8%; while the genes related to stress response were P1: 2%; P2: 2.3%; and P3: 2.1% (Supplementary Table S3).

### Analysis of the Resistome

The data assembly generated contigs with N50 values of 845, 4,240, and 874 for sites P1, P2, and P3, respectively (Table 1). The generated contigs were submitted to the identification of ARGs by CARD. We identified 189, 45, and 123 genes at sites P1, P2, and P3, respectively. Of these, 23.4, 40.9, and 34.2%, respectively, were described as genes that may confer resistance to more than one class of antibiotics.

**FIGURE 5:**
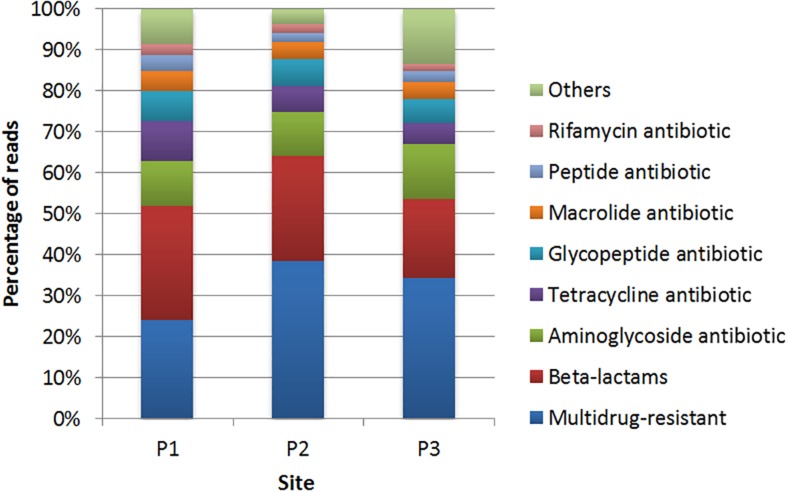
Distribution of the genes conferring resistance to different classes of antibiotics identified by comparison with the CARD database at sites P1, P2, and P3.

The classes of antibiotics associated with the identified genes are shown in Figure 5. The results showed that, in general, genes conferring resistance to the class of β-lactams were the most abundant in all sampling sites, with 29.3, 20.5, and 20% in P1, P2, and P3, respectively (Supplementary Table S4). The *lra-13* gene, encoding a fusion class C/D β-lactamase was the most represented at sites P1 and P3, while the *bla*_TEM_ genes, encoding class A β-lactamases, were identified at all three sites. Genes encoding resistance to aminoglycosides were identified at the three sites. However, there was a higher frequency of these genes at the site P1 in relation to the others sites, with most of the genes identified encode acetyltransferases (AACs) and phosphotransferases (APHs).

Regarding genes associated to resistance to tetracycline, the site P1 showed a higher frequency of genes with high identity in relation to the other two sites. In respect to resistance to glycopeptides, *vanTC*, encoding a membrane-bound serine racemase, was presented at site P2. Genes *vanHF* (encoding a D-specific alpha-ketoacid dehydrogenase) and *vanTG* (encoding a membrane-bound serine racemase) were identified at sites P1 and P3.

In relation to macrolide resistance genes, *mef* (B) (coding for an efflux protein) was identified at sites P1 and P3. Genes encoding resistance to peptide antibiotics represented 3.7% in P1, 2.3% in P2 and 2.5% in P3, with 11 genes predicted, from which the *bcrC* gene, encoding a putative bacitracin transport permease, was predicted at site P1 with high identity. Genes encoding resistance to rifamycin were predicted at sites P1 (2.7%), P2 (2.3%), and P3 (1.7%), and all the predicted genes at sites P2 and P3 were also predicted at site P1. Genes that confer resistance to colistin were also detected, such as *mcr*-5 at sites P1. The category named “others” represents less abundant sequences (Figure 5).

### Isolation and Identification of Strains Resistant to Antibiotics

A total of 98 bacterial isolates were obtained from Lake Bolonha. Of these, 53 isolates were retrieved from culture medium supplemented with cefotaxime (CTX^R^), and 45 were retrieved from medium with imipenem (IMI^R^). Among the sites, P1 presented 21 CTX^R^ isolates and 16 IMI^R^; in P2, 14 CTX^R^ and 12 IMI^R^; and in P3, 18 CTX^R^ and 17 IMI^R^.

The analysis of the 16S rRNA genes indicates that all isolates belonged to the Gammaproteobacteria class, and the most abundant genera were *Acinetobacter*, *Pseudomonas*, *Klebsiella*, and *Enterobacter* (Supplementary Table S5). The genera *Acinetobacter* and *Pseudomonas* were the most abundant in medium with imipenem, with 16 and 13 isolates, respectively. In medium with cefotaxime, the genus *Pseudomonas* was the most abundant, with 24 isolates, followed by the genus *Acinetobacter*, with 16 isolates obtained.

### Analysis of Antibiotic Susceptibility Profiles, ARGs, and Mobile Genetic Elements

**FIGURE 6:**
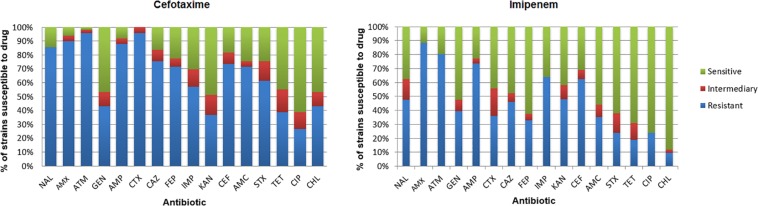
Incidence of resistance to antibiotics among isolates. Amoxicillin (AMX), amoxicillin/clavulanic acid (AMC), ampicillin (AMP), ceftazidime (CAZ), cephalothin (CEF), cefotaxime (CTX), cefepime (FEP), imipenem (IMP), ciprofloxacin (CIP), gentamicin (GEN), chloramphenicol (CHL), sulfamethoxazole/trimethoprim (SXT), kanamycin (KAN), aztreonam (ATM), nalidixic acid (NAL), and tetracycline (TET).

Isolate susceptibility to antibiotics is shown in Figure 6. The resistance to aztreonam (ATM; 96.2%) and resistance to cefotaxime (CTX; 94.3%) were more frequent among the isolates from the medium supplemented with cefotaxime, followed by resistance to amoxicillin (AMX; 90.5%), ampicillin (AMP; 88.6%), nalidixic acid (NAL; 86.7%), cephalothin (CEF; 75.4%), ceftazidime (CAZ; 75.4%), and amoxicillin/clavulanic acid (AMC; 71.6%) (Supplementary Tables S5, S6). Among IMI^R^ isolates, most were resistant to amoxicillin (AMX; 88.5%), aztreonam (ATM; 80.3%), ampicillin (AMP; 73.7%), imipenem (IPM; 63.9%), cephalothin (CEF; 62.3%), kanamycin (KAN; 48.0), nalidixic acid (NAL; 47.6%), and ceftazidime (CAZ; 45.9%). The results demonstrated that among the isolates, 94.9% from cefotaxime-supplemented medium and 85.7% from imipenem-supplemented medium were resistant to three or more classes of antibiotics, making them multidrug resistant.

Regarding isolates retrieved from medium supplemented with cefotaxime, the *bla*_CTX_ gene was detected in 15 isolates (28.3%), the *bla*_SHV_ gene in 12 isolates (22.6%), the *bla*_TEM_ gene in 10 isolates (18.8%), the *bla*_IMP_ gene in eight isolates (15.0%) and the *bla*_VIM_ gene in two isolates (3.7%). Of the isolates retrieved from medium with imipenem, 13 isolates (28.8%) carried the *bla*_VIM_ gene, 10 isolates (22.2%) the *bla*_IMP_ gene, four isolates (8.8%) the *bla*_CTX_ gene, and three isolates (6.6%) the *bla*_KPC_ gene.

## Discussion

In this study, we evaluated the microbiome and resistome in Lake Bolonha. This lake is located in the immediate vicinity of the city of Belém, constituting one of its main sources of drinking water after proper treatment. Urbanization of adjacent areas as well as contamination of rivers that contribute water to the lake could alter the microbiome and resistome, resulting in an increased prevalence of antibiotic resistant bacteria and resistance genes. Since the water is used for human consumption after treatment and for other uses, these changes may pose a significant risk to human and environmental health. Physicochemical parameters were evaluated as water quality meters. The study allowed the description of the microbiome composition and the metagenome was analyzed with special focus on genes related to antibiotic resistance. To complement these results, bacterial strains resistant to antibiotics considered critical to human health were obtained and characterized.

### Bacterial Community Analysis

Lake Bolonha is characterized by the Amazonian vegetation at its margins and by the proliferation of macrophytes under its surface, leading to a process of eutrophication. The elevation of the values of phosphorus and total nitrogen observed in the physical-chemical analysis indicate vegetation decomposition in the lake or the presence of contaminants of human origin. The ability to conduct electricity is related to the concentration of ions present in the medium. Thus, a high electrical conductivity rate may indicate corrosive characteristics of the water caused by industrial evictions and domestic sewage (Hayashi, 2004). Total coliforms were also higher than the reference value. The presence of fecal coliforms is the most appropriate indicator for the presence of fecal contamination, with the potential risk of the presence of pathogenic organisms (Zhurbenko et al., 2017). The pH rate was below the reference value at the three collection points. The change in pH may influence microorganisms in freshwater as an important selection factor and is one of the environmental variables strongly related to the distribution of bacterial groups (Lindström et al., 2005). Although it is an ecological protected area, there are reports of illegal houses on the lakeshore and of illegal use of the lake water. The results obtained for the characterization of water quality are consistent with these anthropogenic pressures.

The analysis of the bacterial community detected the following most abundant phyla: Proteobacteria, Actinobacteria, Firmicutes, Bacteroidetes, and Cyanobacteria. Corroborating studies of diversity conducted in the Amazon Basin, such as the ones conducted by Toyama et al. (2016) and Santos-Júnior et al. (2017), found these same phyla as the main components of bacterial diversity.

Proteobacteria were the most abundant in all collection sites, with the classes Betaproteobacteria and Alphaproteobacteria being the most represented. These classes are abundant in marine and freshwater ecosystems because they adapt to environments with low nutrient concentrations due to their ability to degrade a variety of organic compounds (Salcher et al., 2013; Salka et al., 2014).

The abundance of *Actinobacteria*, although not exceptional, should be highlighted because bacteria included in this phylum are responsible for the production of approximately 75% of the known bioactive compounds, such as antibiotics, enzyme inhibitors, antitumor agents and antivirals (Farnaes et al., 2014). The genus *Streptomyces* was one of the most represented in our results, corroborating other studies carried out in freshwater environments (Sanasam et al., 2011; Zothan et al., 2015, 2018). Another genus of Actinobacteria detected in our study was *Mycobacterium*. This genus includes some species of opportunistic pathogens that have already been reported in freshwater environments (Lee et al., 2016; Roguet et al., 2016; Zhang et al., 2017).

Of the Firmicutes classes, Clostridia was most abundant. This class includes bacteria with notorious importance in the area of medical and industrial microbiology (Baldassi, 2005). In the Clostridia class, the most abundant detected genus was *Clostridium*, which includes pathogenic species (Bezirtzoglou et al., 1997).

In our functional analysis, the cluster-based subsystem was the most abundant at the three collection sites. This subsystem encompasses genes found close to each other within the genomes of several taxa, with some genes having functions associated with proteasomes, ribosomes and aggregates related to recombination (Figure 4; Delmont et al., 2012).

The high index of the carbohydrate, amino acid and derivative subsystems indicates that Lake Bolonha has high nutrient contents that can be used in essential biological or structural processes (Lombard et al., 2014).

To identify the functions related to antibiotic resistance, we highlight the subsystem of virulence and defense, which is associated with islands of pathogenicity, resistance to antibiotics and toxic compounds. Bacterial virulence factors may be encoded in mobile genetic elements, which can be disseminated by horizontal gene transfer, leading to the conversion of non-pathogenic bacteria into pathogens (Bhatt et al., 2014). Functions related to ABC multidrug efflux systems, other efflux pumps and class A, B, and C β-lactamases (Supplementary Table S3) were found.

In addition to the virulence and defense subsystem, stands out the stress response subsystem. Bacteria confront many stresses in natural environments, and these can cause adaptive responses in bacterial cell physiology that, furthermore to protecting against adverse conditions, act on antimicrobial susceptibility (Poole, 2012; Dam et al., 2018). Among the results obtained, there was a higher relation to oxidative stress in the three sites. Reactive oxygen species, by-products of aerobic respiration, elicit adaptive responses to oxidative stress, such as the expression of multiple multi-drug efflux systems (Poole, 2012). In addition, a study demonstrated that the production of indole protein, an extracellular signaling molecule synthesized as a response to oxidative stress, increases the antimicrobial resistance of *P. aeruginosa* (Lee et al., 2009).

### Analysis of the Resistome

Our results identified genes associated with the virulence and defense subsystem, which have the potential to confer resistance to more than one class of antibiotics, as is the case for genes encoding efflux pumps. The prevalence of these genes in Lake Bolonha, which, although belonging to a protected area, is located in the metropolitan area of the city of Belém, may be an indication of anthropic pressure.

The prevalence of genes encoding resistance to β-lactams is worrisome, since these antibiotics are used to treat serious human infections. Genes detected in our analysis were also reported in other studies performed in wastewater, in which β-lactam resistance genes were the most abundant (Smith et al., 2004; Jiang et al., 2013). For instance, the *bla*_TEM_ genes, detected at the three sampling sites, code for one of the most common β-lactamase families in Gram-negative bacteria (Ojdana et al., 2014). This family includes enzymes that confer resistance to penicillins and 1st- and 2nd-generation cephalosporins but also to extended-spectrum β-lactamases (ESBLs) of high clinical relevance (Bradford, 2001; Worthington and Melander, 2014). Of note was the occurrence of genes that are generally associated with mobile genetic elements such as the *bla*_IMP_ (P1 and P3 sites) and *bla*_VIM_ genes (P3 sites) (Marques et al., 2015). These genes encode resistance to carbapenems, a group of antibiotics commonly used as a last resort for the treatment of serious infections (Tacão et al., 2015). At the P3 site, we also detected the *bla*_CTX–M_ gene, which encodes ESBLs whose occurrence in the lake Água Preta, adjacent to Lake Bolonha, has also been recently reported (Freitas et al., 2019).

Due to the indiscriminate use of tetracyclines, tetracycline resistance genes are the most frequently detected ARGs in aquatic environments (Chopra and Roberts, 2001). In addition, genes from the *tet* family have been described in several environments, including effluents, and these are associated with the most common mechanisms of resistance, such as inactivation by chemical drug modification, efflux, and release of ribosome-bound antibiotics (Macauley et al., 2007).

The P3 site appears to have particular characteristics in terms of the resistome. In addition to the genes already mentioned above (i.e., *bla*_IMP_, *bla*_VIM_ and *bla*_CTX–M_), other clinically relevant genes have also been detected at this site, such as *mcr*-5 genes, which confer resistance to colistin and which have recently been detected in plasmids from environmental, human and animal derived isolates (Sun et al., 2017; Tacão et al., 2017; Chen et al., 2018). Furthermore, in this site, the diversity of resistance genes found was superior to that of the other two sites (Supplementary Table S4).

The presence of antibiotics in aquatic environments has already been reported in reservoirs of water, rivers, lakes and even in drinking water after treatment. Even at low concentrations, antibiotics may select for antibiotic resistance, contributing to the evolution and spread of ARGs. The antibiotics present in the water can come from sources such as agriculture, the pharmaceutical industry and hospitals (Kolpin et al., 2004; Valcárcel et al., 2013; Sui et al., 2015). Although there are no reports in the literature confirming the dumping of contaminated water from these sources into Lake Bolonha, the identification of genes associated with antibiotic resistance in Lake Bolonha may be an indication of the presence of antibiotics in this lake. Other contaminants, such as metals and biocides, have been described as selectors of antibiotic resistance (Pal et al., 2017; Romero et al., 2017). However, the concentrations of antibiotics, metals and biocides should have been determined to confirm the presence of these contaminants in Lake Bolonha. On the other hand, we cannot exclude a contribution by environmental sources. In fact, the occurrence of antibiotic producers (e.g., *Streptomyces* strains) in this environment probably led to the selection of antibiotic resistance mechanisms among these producing strains and other microorganisms that share the same ecological niche, thus enriching the lake resistome.

### Strains Resistant to Antibiotics

Analysis of the 16S rRNA gene revealed that most of the selected isolates belonged to the genera *Acinetobacter*, *Pseudomonas*, *Klebsiella*, and *Enterobacter*. These genera are found in aquatic environments and encompass some opportunistic pathogens (Manchanda et al., 2010; Vaz-Moreira et al., 2012). *Klebsiella* has been detected in surface water samples and is frequently identified as *K. pneumoniae*, which is a well-known Gram-negative pathogen against which β-lactam antibiotics are frequently used (Podschun et al., 2001). Regarding *Pseudomonas* spp. isolated from aquatic environments, studies suggest that multiresistance can be acquired and persist even in *Pseudomonas* species that normally are not in direct contact with humans. This genus is considered a reservoir of ARGs, some of which may spread through horizontal gene transfer (Kittinger et al., 2016; Teixeira et al., 2016).

In the susceptibility analysis, a high prevalence of β-lactam antibiotic resistance was observed. This result was expected, since in order to obtain these isolates the samples were cultivated in medium supplemented with cefotaxime and imipenem. β-Lactam resistance is generally related to the production of β-lactamases, enzymes capable of hydrolyzing β-lactam antibiotics by hydrolyzing the amide bond of the β-lactam ring (Topaloglu et al., 2010; Bonomo, 2016). These enzymes are frequently encoded in plasmids and are related to the propagation of resistance among Gram-negative bacteria (Bush and Jacoby, 2010). A single β-lactamase may confer resistance to several β-lactams (Ranjbar and Sami, 2017), which explains the high resistance rate of the isolates to these antibiotics.

*bla*_CTX–M_, as well as some *bla*_SHV_ and *bla*_TEM_ genes, encode ESBLs, one of the largest and most clinically relevant β-lactamase groups (Bush, 2010). *bla*_CTX–M_ was the most abundant among isolates selected from medium supplemented with cefotaxime, which is in accordance with the hydrolytic profile of these enzymes (Shahid et al., 2011). *bla*_TEM_ and *bla*_SHV_ are frequently found in *Enterobacteriaceae* species, for example, in *E. coli* and *K. pneumoniae* (Bradford, 2001). A study conducted to correlate the *bla*_TEM_ gene with cultured bacteria, antimicrobial residues and the composition and structure of the bacterial community in wastewater suggested this gene as one of the indicators to monitor resistance in environmental samples (Narciso-Da-Rocha et al., 2014).

Despite the efficiency of ESBLs in conferring resistance to third- and fourth-generation cephalosporins and monobactams, they have little activity against carbapenems, which are considered the last choice for the treatment of infections caused by multidrug resistant bacteria (Bush, 2010). However, the increased use of carbapenem antibiotics has contributed to the increasing resistance rates reported in clinical isolates. Carbapenemases are the most common mechanism of resistance to these antibiotics, posing serious threats to human health (Yang et al., 2016). Therefore, we sought to identify genes encoding some of the clinically significant carbapenemases, namely, IMP, VIM and KPC, encoded by the *bla*_IMP_, *bla*_VIM_ and *bla*_KPC_ genes, respectively (Moosavian and Rahimzadeh, 2015), some of which had been detected through metagenomics.

As expected, these genes were more common in isolates retrieved from medium supplemented with imipenem, with the *bla*_VIM_ gene being the most abundant, followed by the *bla*_IMP_ gene. The presence of these genes has also been reported in other aquatic environments (Igbinosa et al., 2014; Tacão et al., 2015; Ye et al., 2018). The *bla*_KPC_ gene was also detected, although less frequently. This gene has been reported mainly in *K. pneumoniae* and *E. coli* and is considered a health problem worldwide (Shanmugam et al., 2013). Its occurrence in aquatic systems has been reported (Yang et al., 2016; Sun et al., 2017).

Moreover, in the results it was observed that eight strains of *Acinetobacter*, three strains of *Klebsiella* and fourteen strains of *Pseudomonas*, which were resistant to imipenem, did not amplify the genes *bla*_VIM_, *bla*_IMP_ and *bla*_KPC_. This result is possibly related to the presence of other carbapenemase genes in these strains, such as *bla*_OXA–48_ and *bla*_NDM_, which were not the target of the present study. In the metagenome data the main described OXA groups were not found, such as OXA-48, OXA-23, OXA-40, and OXA-58. However, other genes such as *bla*_OXA–9_, *bla*_OXA–12_, *bla*_OXA–29_, *bla*_OXA–50_, *bla*_OXA–60_, *bla*_OXA–196_, and *bla*_OXA–211_ were found. Thus, these strains may have some other gene that confers resistance to imipenem that was not identified in the PCR analysis.

The results from the analysis of the resistome of Lake Bolonha suggest a reservoir of bacteria resistant to antibiotics and resistance genes of great importance. Our detection of genes conferring resistance to antibiotics of last resort (e.g., carbapenems) should be emphasized. Some of these genes (e.g., *bla*_IMP_ and *bla*_VIM_) were detected both by metagenome analysis and in bacterial isolates. The data were obtained using culture-dependent and -independent technologies, which were complementary and allowed a more robust analysis of the resistome. These results are particularly relevant since the water of this lake is used for human activities, including as a domestic water supply. Thus, the information obtained can serve as a database for the formulation of measures related to sanitation and health in this area.

## Data Availability Statement

Publicly available datasets were analyzed in this study. This data can be found here: https://www.ncbi.nlm.nih.gov, https://www.ncbi.nlm.nih.gov/sra/PRJNA506429.

## Author Contributions

JA, AS, IH, and AF conceived the study. JA and LD performed the collection and processed the samples. JA, JL, and LS performed PCR-DGGE, PCA, and PERMANOVA analyses. JA and DG analyzed the diversity of the community. JA and RR performed the analysis of the resistome. JA and AF identified the strains by 16S rRNA gene sequencing. JA, LD, JaM, and JoM performed the analysis of the resistance profile of the isolates. JA, LD, and IH wrote the manuscript in consultation with all other authors.

## Conflict of Interest

The authors declare that the research was conducted in the absence of any commercial or financial relationships that could be construed as a potential conflict of interest.

## Footnotes

1 http://www.bioinformatics.babraham.ac.uk/

2 http://hannonlab.cshl.edu/fastx_toolkit/

3 https://blast.ncbi.nlm.nih.gov
