# Supplementary figures and images for: Resistome in Lake Bolonha, Brazilian Amazon: Identification of Genes Related to Resistance to Broad-Spectrum Antibiotics
Source: Front Microbiol. 2020 Feb 4;11:67. doi: 10.3389/fmicb.2020.00067 (PMC7010645; doi:10.3389/fmicb.2020.00067)

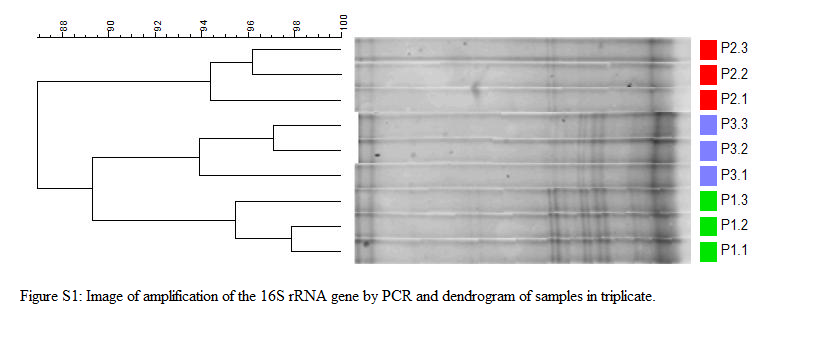

Supplement: Supplementary file 1 [file Image_1.tif]

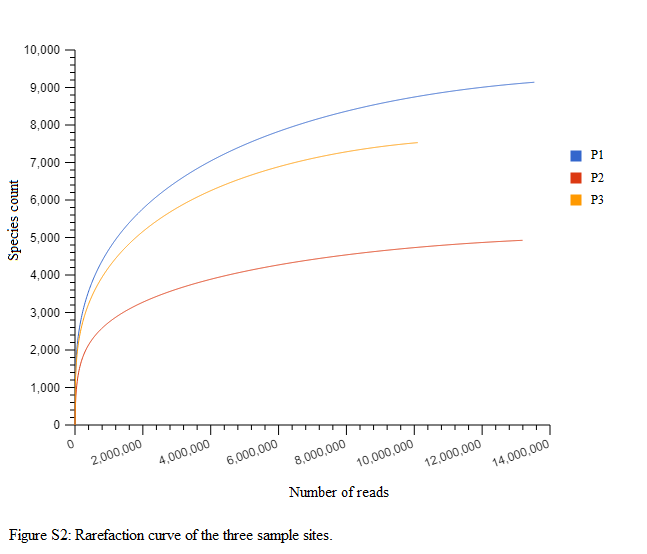

Supplement: Supplementary file 2 [file Image_2.tiff]

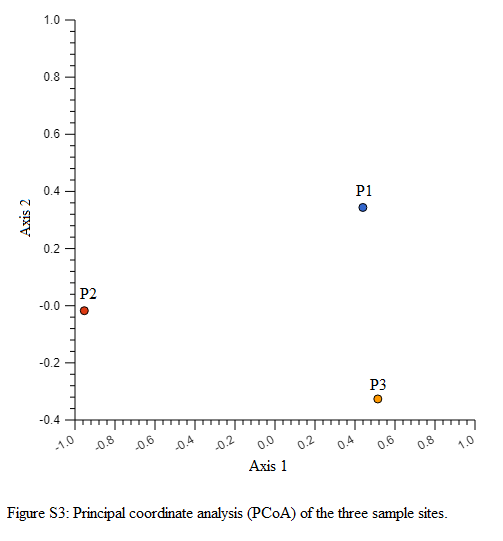

Supplement: Supplementary file 3 [file Image_3.tif]

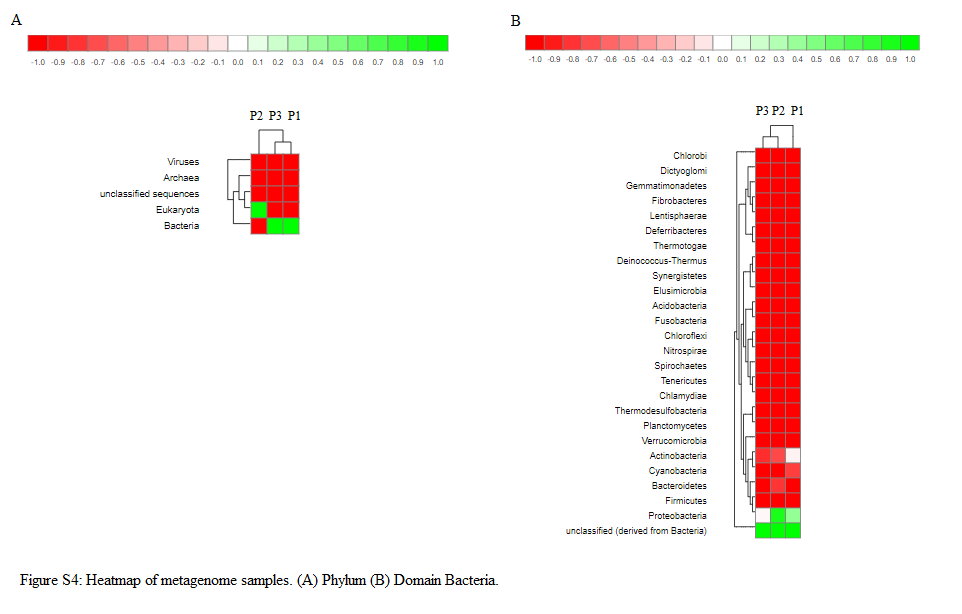

Supplement: Supplementary file 4 [file Image_4.tif]
